# Combined diagnostic accuracy of two artificial intelligence systems for glaucoma diagnosis using color fundus photography

**DOI:** 10.1371/journal.pone.0349878

**Published:** 2026-05-28

**Authors:** Gilvan Vilarinho da Silva Filho, Gustavo Coelho Caiado, Felipe Zocatelli Yamamoto, Sergio Henrique Teixeira, Tiago dos Santos Prata, Carolina Pelegrini Barbosa Gracitelli, Augusto Paranhos Jr

**Affiliations:** Department of Ophthalmology and Visual Science, Glaucoma Service, Universidade Federal de São Paulo, São Paulo, Brazil; Isra Private University: Isra University, JORDAN

## Abstract

**Purpose:**

To evaluate the diagnostic accuracy of two commercially available artificial intelligence (AI) systems based on color fundus photography (CFP), the Laguna ONhE and VUNO Med-Fundus AI, for glaucoma detection, both independently and in combination.

**Methods:**

This retrospective cross-sectional study included 370 eyes from 193 patients (248 eyes with primary open-angle glaucoma and 122 healthy eyes). All eyes underwent structural evaluation with swept-source optical coherence tomography (Triton, Topcon) and visual field testing (Octopus 900, Haag-Streit AG). Fundus photographs were analyzed using Laguna ONhE and VUNO Med-Fundus AI systems. Diagnostic accuracy was evaluated.

**Results:**

Both AI systems demonstrated high diagnostic accuracy. Laguna achieved an AUC of 0.879 using Glaucoma Discriminant Function (GDF), and VUNO showed an AUC of 0.857. When combined, GDF + VUNO achieved an AUC of 0.903. The Global Mean Deviation (GMD) reached the highest diagnostic accuracy (AUC = 0.916), which was not significantly different from GDF + VUNO (p = 0.146).

**Conclusions:**

Laguna ONhE and VUNO Med-Fundus AI had high diagnostic accuracy in detecting glaucoma using only CFP. Their combined use improved further, achieving accuracy comparable to the GMD. This represents a practical approach for glaucoma screening, particularly in settings without access to OCT or automated perimetry.

## Introduction

Glaucoma is the leading cause of irreversible blindness in the world, affecting more than 66 million individuals worldwide [1]. Glaucoma prevalence tends to increase over the years due to the aging of the population [2]. The number of people with glaucoma in the world is expected to increase from 64.3 million in 2013 to 111.8 million in 2040 [2]. As it is a serious progressive disease, early diagnosis is essential. Around 50% of glaucoma patients remain undiagnosed worldwide [3].

The diagnosis of glaucoma is based on the structural (optic nerve) and functional (visual field) assessment of the patient [4]. The optic nerve can be evaluated using fundoscopy, color fundus photography (CFP) and optical coherence tomography (OCT) [4]. Structural analysis of the optic nerve head (ONH) using OCT is a well-established method in the literature. Numerous studies have already shown its importance in the diagnosis of glaucoma, as well as in detecting disease progression [5–9]. However, OCT devices are relatively expensive and not widely available to the general population, which limits their use in large-scale screening programs [10,11].

CFP is a noninvasive and widely available imaging modality that provides structural information about the retina and ONH. In this scenario, automated image analysis tools based on artificial intelligence (AI) have emerged as low-cost and accessible alternatives for ONH evaluation [12–14]. Among these, two software platforms have gained attention: Laguna Optic Nerve Head Hemoglobin Estimation (ONhE) and Vuno Med-Fundus AI (Vuno Inc., Seoul, South Korea).

The Laguna ONhE software is a fully automated colorimetric analysis system, developed by de la Rosa et al. in 2013, that estimates the relative amount of hemoglobin (Hb) within the ONH using red-green reflectance ratios normalized to retinal vessel, where the percentage of Hb is 100% [15,16]. This program generates indices such as the Glaucoma Discriminant Function (GDF), which integrates Hb gradients between central and peripheral regions, showing good correlation with both structural and functional parameters [15,16]. Subsequent refinements introduced deep learning-based ONH segmentation to enhance reproducibility and automate analysis [17–19]. Previous studies have demonstrated a good reproducibility of the Laguna ONhE software, as well as a good accuracy in the diagnosis of mild, moderate and advanced glaucoma [15,16,20–24].

The VUNO Med-Fundus AI system was developed in collaboration with the Seoul National University Bundang Hospital (SNUBH) to assist in the automated detection of retinal and optic nerve diseases [25,26]. Using a large dataset of CFP annotated by ophthalmologists, the algorithm employs convolutional neural networks (CNNs) trained to identify optic disc and retina abnormalities [27–29]. This model achieved an AUC of 99.5 (95% CI, 96.1–100.0) for glaucoma detection when compared with an expert-annotated dataset comprising more than 100,000 CFP from the SNUBH Retina Archive [30].

Due to their low cost and accessibility, Laguna ONhE and VUNO Med-Fundus AI represent promising tools for large-scale glaucoma screening based on CFP, which motivated the present research. The purpose of this study was to evaluate the diagnostic accuracy of these two AI tools, trained on different datasets, for glaucoma detection using CFP, both independently and in combination.

## Methods

This retrospective cross-sectional clinical study included 469 eyes from 243 patients (181 patients with primary open-angle glaucoma and 62 healthy controls) evaluated between 2017 and 2025 at Clínica Forno e Paranhos (São Paulo, Brazil) and Hospital de Olhos Previsão (Teresina, Brazil). Of these, 370 eyes from 193 participants were ultimately included in the final analysis after exclusion of eyes with poor OCT signal quality or unsuccessful Laguna ONhE analysis. The sample size was determined by the number of eligible participants available in the clinical databases during the study period. The higher proportion of glaucoma eyes reflects the retrospective design and the fact that both participating centers are glaucoma referral clinics. Data were accessed between June 30, 2024 and June 30, 2025.

The study was approved by the Ethics Committee of Universidade Federal de São Paulo (approval number: 66432522.0.1001.5505), with a waiver of informed consent due to its retrospective nature.

### Participant selection

Eligible participants were required to have a best corrected visual acuity (BCVA) ≥0.1 on the decimal scale, open angle in gonioscopy, and no ocular or systemic comorbidities capable of affecting the visual field (VF) or OCT and retinography quality. Additional inclusion criteria included: absence of retinal alteration or opacity that could compromise the VF, absence of ocular disease except glaucoma, no history of ocular surgery other than antiglaucoma surgery and uncomplicated cataract surgery, a spherical refractive error between -10D and +6D, and astigmatism ≤ 2D.

Glaucoma was defined as an optic neuropathy characterized by typical structural damage to ONH, with corresponding loss of the peripapillary retinal nerve fiber layer (pRNFL) and the macular ganglion cell complex (GLC++) on OCT, with or without corresponding VF defects. Therefore, preperimetric glaucoma cases were also included in this study. Healthy subjects required intraocular pressure (IOP) <21mmHg, a normal ONH appearance on fundoscopy, and normal OCT and VF findings.

Exclusion criteria included BCVA <0.1, diabetic retinopathy, previous retinal vein occlusion, macular degeneration, amblyopia, epiretinal membrane, ONH or VF abnormalities of neurologic origin, use of systemic medications that may affect the retinal vasculature, patients with unreliable VF (false positives ≥15%, false negatives ≥15%, or presence of artifacts), low-quality OCT scans, and fundus images with opacities that may impair the Laguna ONhE or the VUNO Med-Fundus AI analysis.

All included participants underwent a comprehensive ophthalmologic evaluation including detailed medical and family history, visual acuity measurement, slit-lamp biomicroscopy, gonioscopy, fundus examination and IOP assessment on the same day.

### Visual field assessment

VF testing was performed using the Octopus 900 (Haag-Streit AG, Koeniz-Berne, Switzerland) with Goldmann size III stimuli, the Tendency Oriented Perimeter (TOP) strategy, and the G program (which includes 59 test locations within the central 30 degrees). Only eyes with at least two reliable VF tests (false positives <15%, false negatives <15% and no artifacts) were included.

Glaucomatous VF defects were defined as reproducible abnormalities consistent with glaucoma and not attributable to other ocular or neurological conditions. All VF tests were reviewed and classified by a glaucoma specialist.

### CFP and OCT assessment

For the structural evaluation, all participants underwent CFP and OCT, using the Swept-Source DRI OCT (Triton, Topcon, Tokyo, Japan). In the OCT analysis, the pRNFL and the GLC++ were evaluated using the 3D Wide scanning protocol (12x9mm).

Only images with high signal quality (>40) were included in the analysis.

### Laguna ONhE assessment

CFP was analyzed using the Laguna ONhE software version 8.0, available on the website http://test.laguna.insoft.es/. This software has been previously described in detail by de la Rosa [15].

In summary, this program uses mathematical algorithms for automatic component segmentation to identify two areas of the ONH: the central retinal vessels and the ONH tissue. Through an analysis program that uses the Matlab image processing toolbox (The MathWorks, Inc., Natick, MA), this software analyzes three spectral components of ONH images: red, green and blue [15]. The idea is that areas with high Hb content (such as neuroretinal rim and vessels) reflect more red light than areas with low Hb content (such as optic cup). Previous studies have shown that it is possible to estimate the amount of tissue Hb using formulas that use these three light spectral components [15]. The presence of hemoglobin in the tissue was estimated by Laguna ONhE in percentage, using the color of the great vessels as a reference (where the percentage of hemoglobin is 100%) [31].

**Fig 1:**
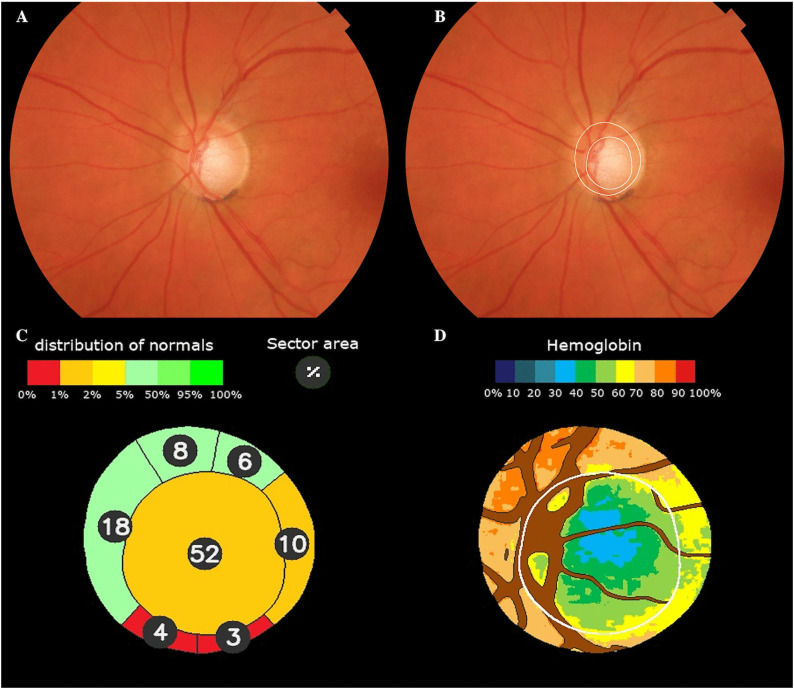
Laguna ONhE Assessment. Example of Laguna ONhE analysis: (A) Original eye fundus image. (B) Automatic segmentation of the optic disc and cup boundaries. (C) Sectoral areas are expressed as a percentage of the optic disc area and compared with values from normative reference population. (D) Segmentation of reference vessels and pseudo-color mapping of hemoglobin distribution.

When uploading the retinography, the software automatically delimits the disc and the cup and also divides the ONH in sectors. An estimate of cup size and position was also obtained, and the results of the cup, rim sectors, vertical cup/disc ratio and cup/disc area ratio were compared with the percentiles achieved in the normal population (Fig 1) [22].

The ONH segmentation was improved using a previously described neural network, trained using deep learning U-Net architecture, that identifies the inner edge of Elsching’s scleral ring [17,18]. This deep learning neural network was also used to identify the eye’s laterality, image quality, vessel segmentation and to exclude abnormal optic discs [31,32].

The distribution of hemoglobin in the inferior and superior zones of the ONH relative to the nasal and temporal zones, together with the relationships between the size and shape of the cupping (weighted according to ONH size) generates the Laguna’s main index: the Glaucoma Discriminant Function (GDF).

The GDF index combines the Hb slope (obtained through multiple regression analysis of Hb estimated for each sector) with the mean Hb in sectors 8 and 20 [15]. This index is expressed as a whole number from −100 to +30 (version 8.0), in which the value 0 was adjusted to correspond to an approximate specificity of 95%. The calculation of the GDF index also incorporates a classifier based on Deep Learning (DL), that generates values between 0 (glaucoma) and 1 (normal) [33]. Therefore, positive GDF values indicate ONH Hb levels within the normal range, whereas negative values indicate a Hb distribution more consistent with glaucoma [15].

A combined index, Global Mean Deviation (GMD), was also generated using data related to morphology (rim volume), perfusion (GDF) and VF parameters (Mean Defect and Square Root of Loss Variance). Unlike GDF, which is derived exclusively from CFP, GMD also requires VF information and therefore cannot be obtained in settings where perimetry is unavailable.

Images with poor quality or with a sectioned or missing optic disc were excluded from the analysis. When Laguna ONhE was unable to analyze a CFP, the software automatically generated an exclusion output indicating the reason for unsuccessful analysis, including “Disc not detected”, “Erroneous disc shape”, “Low image quality” and “Saturated image”.

### VUNO assessment

After being analyzed by the Laguna ONhE software, all CFP were subsequently evaluated using the Vuno Med-Fundus AI platform (available on the website https://fundusai-eu.vunomed.com/login). This program employs deep CNNs trained on large-scale ophthalmologist-annotated datasets from SNUBH to automatically detect retinal and optic disc abnormalities consistent with glaucoma [25–29].

**Fig 2:**
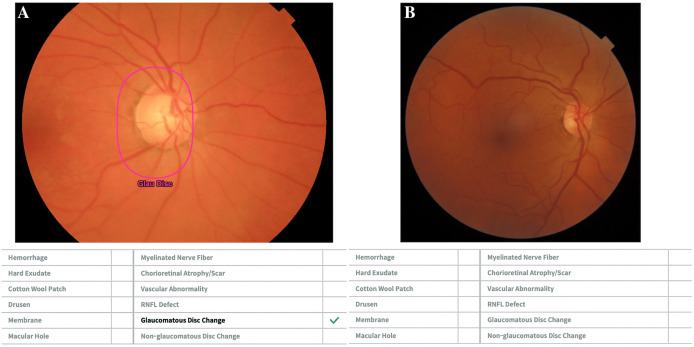
VUNO Assessment. Example of VUNO analysis: (A) Eye classified as glaucomatous. (B) Eye classified as healthy.

The algorithm was trained using over 300,000 retinal fundus images from SNUBH Retina Image Archive. Expert ophthalmologists annotated each image for 12 major retina and optic nerve fin.dings. To improve transparency, VUNO integrated Gradient-weighted Class Activation Mapping (Grad-CAM) to highlight the retina regions contributing most to the decision process. These AI features enable ophthalmologists to visually validate the system’s focus on the optic disc and RNFL regions (Fig 2) [30].

VUNO was validated using internal and external datasets, including Retinal Fundus Glaucoma Challenge (REFUGE) and Indian Diabetic Retinopathy Image Dataset (IDRiD), which provided standardized benchmarks for glaucoma classification and optic disc/cup segmentation [30,34,35].

After uploading the CFP to the software and automatic image processing, each eye was classified according to the VUNO Med-Fundus AI output. Analyses were performed using VUNO Med-Fundus AI model VN-M-03 (VUNO Inc., Seoul, South Korea; software release date: August 16, 2022). Fundus images were analyzed in the VUNO platform between June 2024 and June 2025. Eyes presenting the finding “Glaucoma Disc Change” in the report were categorized as glaucomatous (Fig 2A), whereas those without this finding were classified as controls, even if other abnormalities were detected (Fig 2B). Therefore, VUNO was entered into the statistical models as a binary variable based on the presence or absence of “Glaucoma Disc Change”.

Both Laguna ONhE and VUNO Med-Fundus AI require only a standard computer with internet access, and the processing time for each CFP is typically a few seconds.

### Statistical analysis

Continuous variables were summarized as mean, standard deviation (SD), median and range, while categorical variables were expressed as absolute frequencies and percentages. Binary logistic regression models adjusted for age were fitted separately for each diagnostic parameter, using generalized estimation equations (GEE) to account for inter-eye correlation and the age difference between glaucoma and control groups (p = 0.004). In these models, glaucoma status was defined as the dependent variable, and either the GDF or the VUNO output served as the independent variable. GDF was entered as a continuous variable, whereas the VUNO output was entered as a binary variable based on the presence or absence of the report finding “Glaucoma Disc Change”. GEE was selected because glaucoma is a bilateral but often asymmetrical disease, so randomizing one eye per subject could bias the results by selecting the less affected eye [36,37]. The fitted models were then used for analysis.

The following parameters were evaluated: GDF, VUNO, GMD, and GDF + VUNO. Separate age-adjusted GEE logistic regression models were fitted for GDF and VUNO, and predicted probabilities from these models were extracted. Principal component analysis (PCA) was subsequently applied to these predicted probabilities, and the first principal component was retained as a continuous combined parameter (GDF + VUNO). PCA was selected because it provides an objective method for integrating information from both systems without imposing predefined weights or thresholds [38].

Receiver Operating Characteristic (ROC) curve and areas under the curve (AUC) were subsequently generated using the predicted probabilities derived from the fitted models. The 95% confidence intervals were adjusted using bootstrap per patient cluster (B = 2000), correcting for the dependence between the two eyes. AUC differences were calculated by matching the models in the same bootstrap replicates (same patients in each replicate), and the p-values of the multiple comparisons were adjusted using the Benjamini–Hochberg procedure to control for False Discovery Rate (FDR).

In addition to the AUC and corresponding Youden Index, clinically interpretable measures including sensitivity, specificity, positive predictive value (PPV), and negative predictive value (NPV) were calculated using the optimal cutoff defined by the maximum Youden Index for each model.

An additional subgroup analysis was performed according to pRNFL thickness using the sample median (87.1 µm) as the cutoff. Sensitivity and specificity were calculated separately for eyes with pRNFL ≤87.1 µm and >87.1 µm to explore model performance according to glaucoma severity.

All statistical analyses were performed using IBM SPSS Statistics 30.0.0.0 (IBM Corp., North Castle, USA) and R Studio 2024.04.2 + 764 (2024.04.2 + 764). A p-value <0.05 was considered statistically significant.

## Results

A total of 243 participants (469 eyes), including 181 patients with glaucoma and 62 healthy controls, were initially screened for inclusion. After excluding 13 eyes due to low OCT signal strength (SS < 40) and 86 eyes due to unsuccessful analysis by the Laguna ONhE software, the final sample consisted of 193 participants (370 eyes).

**Fig 3:**
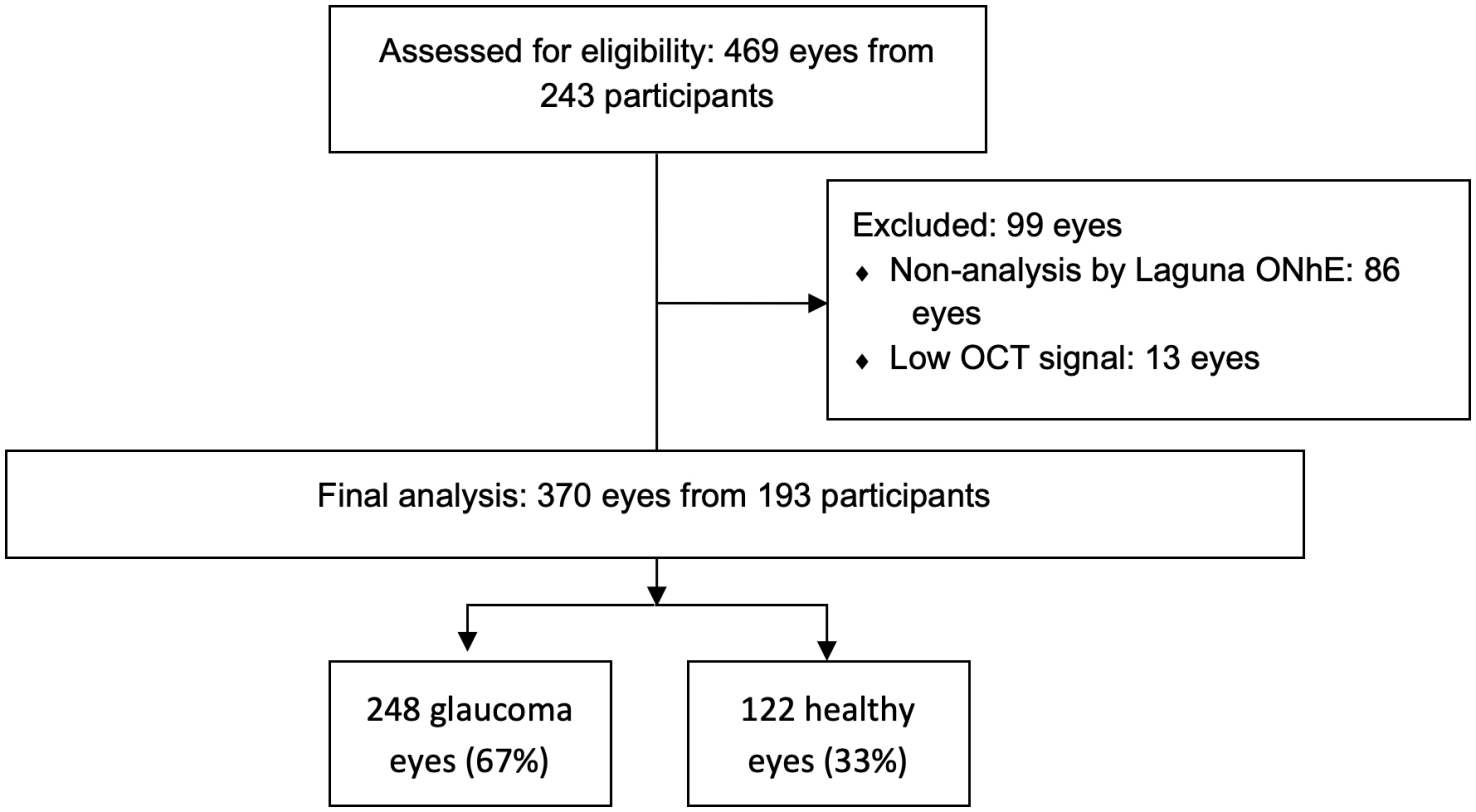
Flowchart of Study Population Selection and Inclusion.

Of the 86 eyes excluded due to unsuccessful Laguna ONhE analysis, the main reasons were “Erroneous disc shape” (41 eyes), “Saturated image” (23 eyes), “Low image quality” (19 eyes), and “Disc not detected” (3 eyes). All images successfully analyzed by Laguna ONhE were also evaluated by VUNO (Fig 3).

A total of 370 eyes from 193 participants (mean age = 63.33 ± 13.98) were included in the final analysis, of which 248 eyes (67%) with primary open-angle glaucoma (POAG) and 122 healthy eyes (33%) (Fig 3). Patients with POAG were significantly older than controls (68.9 ± 15.4 versus 64.3 ± 6.6 years; p = 0.004). The remaining demographics and ocular characteristics of both groups are presented in Tables 1–3. Due to the age difference between the two groups, we used GEE with binary logistic regression to adjust for inter-eye dependence and age. Model-based predicted probabilities were then used to construct ROC curves and estimate AUCs [36].

**Table 1:** Sample Characteristics.

Variable	Healthy eyes (n = 122 eyes)		Glaucoma eyes (n = 248 eyes)	Total Sample
Right eye (n, %)	60 (49.2%)		131 (52.8%)	191 (51.6%)
Left eye (n, %)	62 (50.8%)		117 (47.2%)	179 (48.4%)
Female (n, %)	60 (49.2%)		126 (50.8%)	186 (50.3%)
Male (n, %)	62 (50.8%)		122 (49.2%)	184 (49.7%)
Age, mean ± SD (years)	64.3 ± 6.6		68.9 ± 15.4	63.33 ± 13.98
Age, minimum–maximum (years)	46–78		25–102	25–102

Abbreviations: SD: standard deviation.

**Table 2:** Clinical Characteristics of Healthy Eyes.

Parameter	Eye	N	Mean	Median	SD	Min	Max
MD (dB)	R	60	1.64	1.26	2.09	−2.02	8.05
	L	62	1.72	1.73	1.95	−1.88	8.46
sLV (dB)	R	60	2.26	2.12	0.846	0.67	5.12
	L	62	2.29	2.12	0.822	0.87	4.77
pRNFL (µm)	R	60	105	106	12.3	63.6	136
	L	62	107	107	11.3	77.0	132
GDF	R	60	4.99	13.0	19.0	−52.0	25.8
	L	62	2.53	7.34	19.6	−43.5	28.4
GMD	R	60	−4.58	−2.21	9.15	−28.4	20.9
	L	62	−5.95	−5.56	8.21	−24.2	5.24

Abbreviations: R: right; L: left; n: number of eyes; MD: mean deviation; sLV: Square Root of Loss Variance; pRNFL: peripapillary retinal nerve fiber layer; GDF: Glaucoma Discriminant Function; GMD: Global Mean Deviation; SD: standard deviation; Min: minimum; Max: maximum.

**Table 3:** Clinical Characteristics of Glaucoma Eyes.

Parameter	Eye	N	Mean	Median	SD	Min	Max
MD (dB)	R	131	6.62	5.01	6.17	−1.62	22.3
	L	117	7.03	4.85	6.71	−1.19	24.9
sLV (dB)	R	131	4.73	4.38	2.29	1.18	11.0
	L	117	4.63	4.26	2.29	1.38	9.53
pRNFL (µm)	R	131	73.1	73.0	20.7	19.8	127
	L	117	71.8	74.0	19.9	28.7	113
GDF	R	131	−41.5	−46.5	32.2	−100	24.1
	L	117	−41.5	−44.0	31.9	−100	28.8
GMD	R	131	−35.1	−35.1	20.0	−88.5	4.06
	L	117	−35.6	−33.0	21.0	−92.2	2.34

Abbreviations: R: right; L: left; n: number of eyes; MD: mean deviation; sLV: Square Root of Loss Variance; pRNFL: peripapillary retinal nerve fiber layer; GDF: Glaucoma Discriminant Function; GMD: Global Mean Deviation; SD: standard deviation; Min: minimum; Max: maximum.

The VUNO Med-Fundus AI system showed high accuracy in distinguishing glaucomatous from healthy eyes. Among the 122 control eyes, the algorithm correctly classified 104 eyes (85.2%) and misclassified 18 eyes (14.8%). These results yielded an AUC of 0.857 (Youden Index = 0.711) (Table 4).

**Table 4:** Diagnostic performance of the Vuno Med-Fundus AI system for glaucoma detection.

Metric	Value
True negatives (correctly classified healthy)	104
False positives (misclassified healthy)	18
True positives (correctly classified glaucoma eyes)	213
False negatives (misclassified glaucoma eyes)	35
Accuracy	0.857
Youden Index	0.711

**Fig 4:**
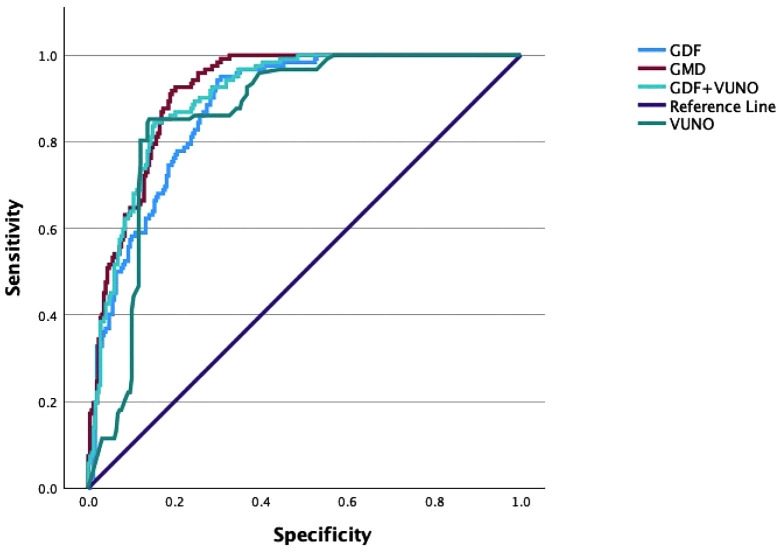
Area under the ROC curve. Abbreviations: ROC, receiver operating characteristic.

All parameters demonstrated high diagnostic accuracy (Fig 4 and Table 5). At the optimal cutoff defined by the maximum Youden Index, sensitivity, specificity, PPV, NPV, and accuracy were additionally calculated for each model to facilitate clinical interpretation of the findings (Table 5).

**Table 5:** Diagnostic performance of parameters.

Parameter	AUC	CI 95%	Youden index	Sensitivity (%)	Specificity (%)	PPV (%)	NPV (%)	Accuracy (%)
GDF	0.879	0.847–0.909	0.644	69.4	95.1	96.6	60.4	77.8
VUNO	0.857	0.827–0.892	0.711	85.9	85.2	92.2	74.8	85.7
GMD	0.916	0.891–0.940	0.725	79.8	92.6	95.7	69.3	84.1
GDF + VUNO	0.903	0.875–0.929	0.691	84.7	84.4	91.7	73.0	84.6

Abbreviations: AUC, area under the curve; CI, confidence interval; PPV, positive predictive value; NPV, negative predictive value.

The GDF model showed the highest specificity (95.1%) and PPV (96.6%), indicating excellent ability to confirm glaucoma when the test result was positive. Meanwhile, the VUNO model demonstrated the highest sensitivity (85.9%) and NPV (74.8%), indicating greater ability to identify glaucomatous eyes and fewer false-negative results. The combined models provided a more balanced profile between sensitivity and specificity, particularly the GMD model, which achieved high specificity (92.6%) and PPV (95.7%) while maintaining good sensitivity (79.8%). The GDF + VUNO model also maintained high sensitivity (84.7%) with overall strong diagnostic performance.

Pairwise comparison of AUCs showed that the GMD achieved the highest diagnostic accuracy (AUC = 0.916, Youden Index = 0.725), significantly outperforming both GDF and VUNO. The difference between GDF + VUNO and GMD was not statistically significant (p = 0.146) (Table 6).

**Table 6:** Comparison of diagnostic performance of parameters.

Parameter	GDF	VUNO	GMD	GDF + VUNO
GDF	—	0.345	<0.001	0.008
VUNO	0.345	—	0.002	0.001
GMD	<0.001	0.002	—	0.146
GDF + VUNO	0.008	0.001	0.146	—

Abbreviations: AUC, area under the curve.

Subgroup analysis according to pRNFL thickness showed higher sensitivity for all models in eyes with lower pRNFL values (pRNFL ≤87.1 µm), whereas performance decreased in eyes with higher pRNFL thickness (>87.1 µm). Specificity remained relatively stable across pRNFL groups (Table 7).

**Table 7:** Diagnostic performance according to pRNFL thickness.

pRNFL (µm)	Model	Sensitivity % (95% CI)	Specificity % (95% CI)
≤87.1 µm	GDF	69.4 (63.2–75.0)	95.1 (89.6–98.2)
	VUNO	85.9 (80.9–90.0)	85.2 (77.7–91.0)
	GMD	79.8 (74.3–84.6)	92.6 (86.5–96.6)
	GDF + VUNO	84.7 (79.6–88.9)	84.4 (76.8–90.4)
>87.1 µm	GDF	18.2 (2.3–51.8)	94.0 (86.5–98.0)
	VUNO	63.6 (30.8–89.1)	84.3 (74.7–91.4)
	GMD	27.3 (6.0–61.0)	91.6 (83.4–96.5)
	GDF + VUNO	36.4 (10.9–69.2)	84.3 (74.7–91.4)

Abbreviations: pRNFL: peripapillary retinal nerve fiber layer; CI, confidence interval.

## Discussion

In the present study, we evaluated and compared the diagnostic accuracy for glaucoma detection of two AI systems based on CFP, Laguna ONhE and VUNO Med-Fundus AI. These tools employ distinct strategies: Laguna ONhE uses a colorimetric approach to estimate hemoglobin content in the ONH, while VUNO Med-Fundus AI uses CNNs to identify patterns suggestive of glaucoma.

Our results demonstrated that both AI systems achieved high diagnostic accuracy when used independently. When combined (GDF + VUNO), their performance improved further (AUC = 0.903), achieving diagnostic accuracy comparable to a single model that uses VF parameters, the GMD provided by Laguna (AUC = 0.916, p = 0.146). This finding indicates that combining two AI analyses based only on CFP can approximate the diagnostic precision obtained by incorporating perimetric information. Since both AI systems used only CFP, a low-cost and accessible method, this approach represents a practical alternative for glaucoma screening, especially in regions with limited access to OCT and automated perimetry.

From a clinical perspective, GDF showed particularly high specificity and PPV, suggesting that a positive result strongly supports the presence of glaucoma. In contrast, VUNO demonstrated higher sensitivity and NPV, which may be advantageous in screening settings where minimizing false-negative results is especially important. Although the combined GDF + VUNO model demonstrated higher overall diagnostic accuracy as measured by AUC, this improvement was not consistently reflected in sensitivity and specificity at the selected cutoff. Instead, the individual models retained more distinct diagnostic characteristics, with GDF favoring specificity and VUNO favoring sensitivity. These findings suggest that the clinical advantage of the combined model may depend on the selected threshold and the intended application, such as screening or confirmatory diagnosis.

Although GMD showed the highest diagnostic performance, it should be interpreted as a composite benchmark rather than an independent external reference, since it incorporates morphology, perfusion, and VF information. Therefore, the finding that GDF + VUNO approached GMD performance should not be interpreted as evidence that the combined model can replace a comprehensive structure-function evaluation, but rather that it may approximate the diagnostic performance of such an evaluation using only CFP-based information.

The combined GDF + VUNO parameter was obtained using PCA, which integrates the continuous outputs of both systems into a single composite variable. This approach does not apply predefined logical rules or classification thresholds, but instead summarizes shared information between the two measures into a continuous score. Accordingly, the improved diagnostic performance observed for the combined model likely reflects the complementary nature of the information provided by each AI system, rather than a rule-based or outcome-driven optimization. Because this method is independent of the glaucoma classification during its construction, it reduces the risk of overfitting and supports the robustness of the combined analysis.

To our knowledge, this is the first study that evaluated the diagnostic accuracy of the GMD index, and the first to integrate the outputs of two commercially available AI systems for glaucoma detection.

The Laguna ONhE system has been extensively validated in the literature for quantifying ONH hemoglobin and its correlation with structural and functional glaucoma parameters. Multiple studies have demonstrated its high reproducibility, accuracy in glaucoma detection (even in mild cases) and good correlation with parameters provided by OCT and VF [15,20,21,23,39–42]. Meanwhile, VUNO Med-Fundus AI employs CNNs capable of detecting glaucomatous disc changes and RNFL defects [25,27,29]. Previous validation studies confirmed its high diagnostic accuracy (AUC > 0.95) in glaucoma datasets [30,35]. Our results are consistent with those reports, since we found good diagnostic accuracy for GDF (AUC = 0.879) and VUNO (AUC = 0.857), reinforcing their clinical utility.

Moreover, our study contributes to this body of evidence by using these two AI systems, each trained on different datasets, in the same population. Their complementary assessment may explain the enhanced diagnostic performance observed when used together (AUC = 0.903). While Laguna ONhE evaluates the vascular integrity of the ONH through hemoglobin distribution [15], VUNO Med-Fundus AI detects structural damage patterns suggestive of glaucoma (ISNT rule violation, rim narrowing/notching) [26].

Recent studies have explored ensemble AI approaches for glaucoma detection. Cho et al. and Kurilová et al. demonstrated that CNNs combining outputs from different architectures had better accuracy than single model designs in glaucoma classification from fundus images, with AUC values exceeding 0.97 [43,44]. Similarly, Sharma et al. proposed a hybrid AI system that combines multiple deep-learning modules to detect glaucoma features (RNFL defects, disc hemorrhages and cup-to-disc abnormalities). These outputs are integrated through a fully connected network, and the final glaucoma probability is refined using image-level predictions, resulting in improved screening performance compared with individual models [45]. These findings corroborate our results, reinforcing that integrating different AI approaches can enhance diagnostic accuracy.

Subgroup analysis according to pRNFL thickness showed that all models performed better in eyes with lower pRNFL values (pRNFL ≤87.1 µm), corresponding to more advanced structural damage. In contrast, sensitivity decreased substantially in eyes with higher pRNFL thickness (>87.1 µm), suggesting lower sensitivity in earlier-stage glaucoma. These findings are consistent with previous studies showing that glaucoma detection becomes easier as disease severity increases, since structural abnormalities become more pronounced in moderate and advanced stages [46–48].

This study has several limitations. Although our dataset included a substantial number of eyes, it was retrospective and derived from two Brazilian glaucoma referral clinics, which may limit generalizability to other ethnic groups or clinical environments. Furthermore, we did not perform external validation in an independent cohort, which is important to confirm the reproducibility of our findings across different populations, although this was not the primary aim of our study.

In addition, the glaucoma group was larger than the control group and included a substantial proportion of moderate and advanced cases, likely reflecting the referral profile of the participating centers. This may have contributed to the high diagnostic performance observed and may not fully reflect a true population-based screening setting. Future research should validate these findings prospectively, incorporate longitudinal data, and determine whether these combined AI systems can predict glaucoma progression or differentiate suspects from true disease more accurately.

Another limitation is that the combined analysis (GDF + VUNO) is not available commercially, as the two AI systems belong to independent companies and are not integrated within a single platform. However, this does not diminish the relevance of our findings, since the combined parameter can be calculated from the individual outputs provided by each software, allowing replication of the methodology whenever both systems are accessible. Future studies may further explore the integration of these AI systems if combined commercial use becomes feasible, including through approaches such as federated learning. Additionally, although the mean age differed statistically between the glaucoma and control groups, the clinical relevance of this difference is small; nonetheless, all models were adjusted for age to avoid potential confounding and ensure that diagnostic performance estimates were not biased by age.

Another important limitation is that both AI systems excluded a subset of eyes from analysis, particularly those with poor image quality or atypical optic disc morphology. This exclusion is inherent to the software and not to the design of our study. As a result, eyes with more challenging ONH assessment – such as those from myopic patients or with peripapillary atrophy – may not be analyzed by the automated platforms. Importantly, these cases were not misclassified as normal, they were simply excluded from analysis, underscoring that additional diagnostic tools remain necessary for eyes in which the AI systems cannot generate reliable outputs.

In conclusion, both AI systems based on CFP, Laguna ONhE and VUNO Med-Fundus AI, demonstrated high diagnostic performance for glaucoma detection, and their combined use further improved diagnostic accuracy. Given their low cost, accessibility, and noninvasive nature, these AI systems may represent a promising strategy for glaucoma detection and triage, particularly in settings where OCT and automated perimetry are not readily available.
